# Supplementary material for: E‐cadherin mediates apical membrane initiation site localisation during de novo polarisation of epithelial cavities
Source: EMBO J. 2022 Aug 22;41(24):e111021. doi: 10.15252/embj.2022111021 (PMC9753465; doi:10.15252/embj.2022111021)
Supplement: Supplementary file 2 — Expanded View Figures PDF [file EMBJ-41-e111021-s001.pdf]

Expanded View Figures

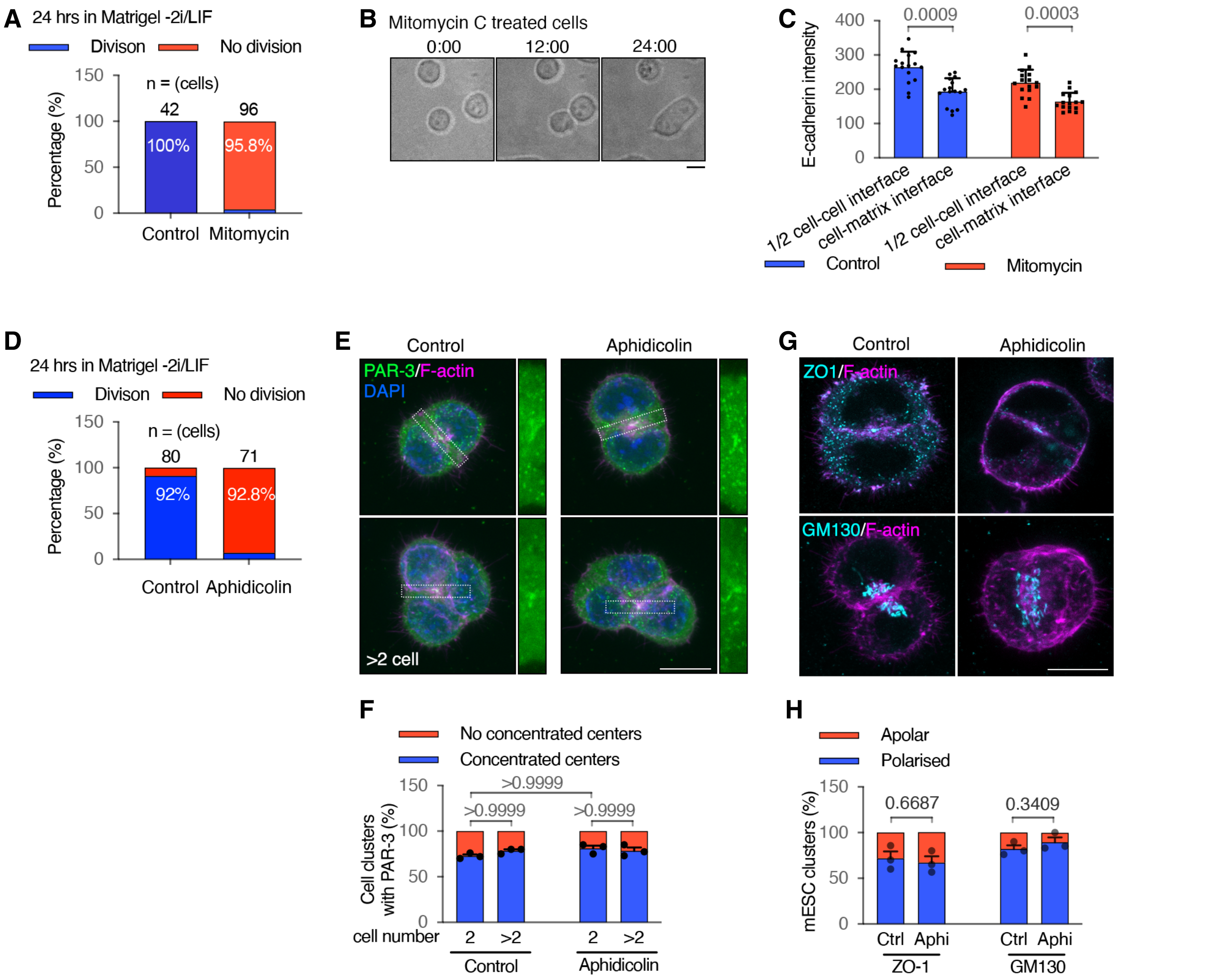

Figure EV1. Division-blocked mESCs in Matrigel 3D cultures.

- A Percentage of cells that did or did not divide in control cells or following mitomycin C treatment. Cell numbers were taken from live movies of the first 24 h following seeding into Matrigel. Mitomycin C sufficiently blocked cell divisions.
- B Movie stills of mitomycin C-treated cells from Movie EV1.
- C Quantification of E-cadherin fluorescence intensity at cell–cell interfaces and cell-matrix interfaces in 2-cell mESC clusters.
- D Percentage of cells that divided or did not divide in control cells or following aphidicolin treatment. Cell numbers were taken from live movies of the first 24 h following seeding into Matrigel. Aphidicolin sufficiently blocked cell divisions.
- E, F Immunofluorescence of PAR-3 (E) and percentages of 2-cell mESC clusters with a positive PAR-3 centre (F) in control and aphidicolin-treated cells cultured for 24 h in Matrigel.
- G, H Immunofluorescence of ZO-1 and Golgi apparatus (G) and percentages of 2-cell mESC clusters with a strong positive ZO-1 centre or polarised Golgi apparatus (H) in control and aphidicolin-treated cells.

Data information: All data are presented as means ± SEM. n = total numbers of cells tracked at time point zero in (A) and (D); 18 cell clusters in each condition in (C); three experiments in (F), (H), 20 clusters were analysed for each column in every experiment. Student's t-test analysis in (C) and (H); two-way ANOVA analysis in (F); P-values were listed in the graphs. All scale bars: 10 μm.
Source data are available online for this figure.

 © 2022 The Authors

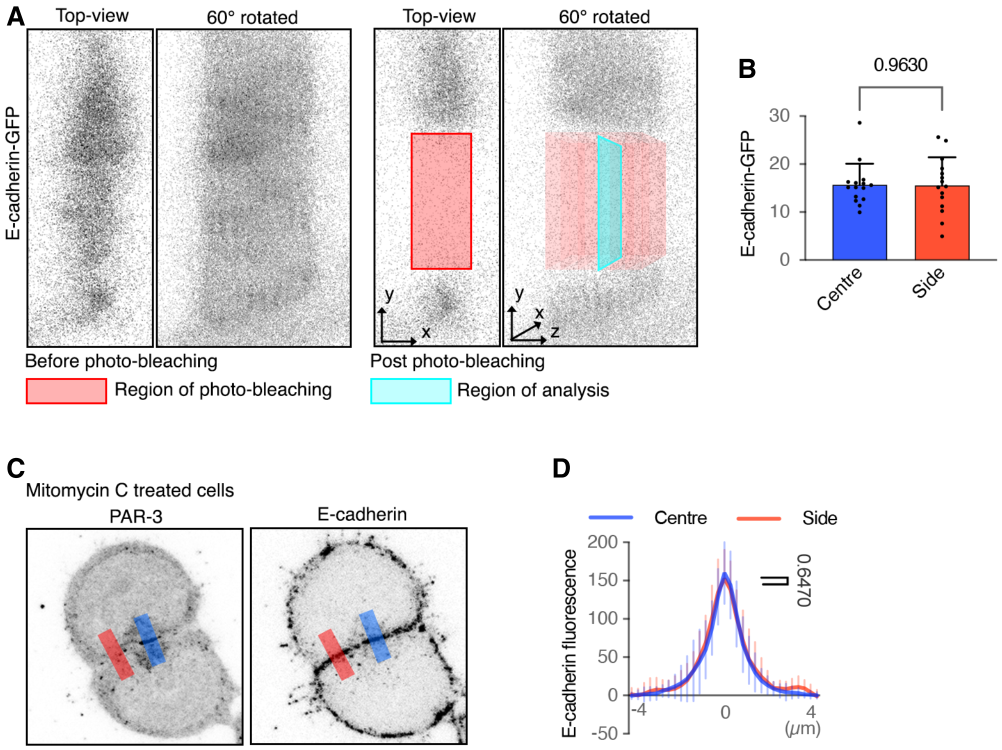

Figure EV2. E-cadherin in the centre-most and side regions at two-cell cluster interfaces.

A Illustrations of E-cadherin-eGFP FRAP at a two-cell cluster interface.

B Average E-cadherin-eGFP pixel levels at the photobleaching regions before bleaching in division-blocked cells.

C An example line-scan at the centre-most (blue) and side (red) regions at division-blocked two-cell cluster interfaces. The width of line-scans was 3 μm. Two side regions were line-scanned, and the average was taken as the line-scan profile at the side regions for a two-cell cluster (See panel B).

D Line-scan profiles of E-cadherin at the centre-most and side regions. The statistical comparison was done between the area under the curves.

Data information: In (B) and (D), data are means ± SD; n = 15 cell clusters; Student's t-test analysis; P-values were listed in the graphs.
Source data are available online for this figure.

© 2022 The Authors

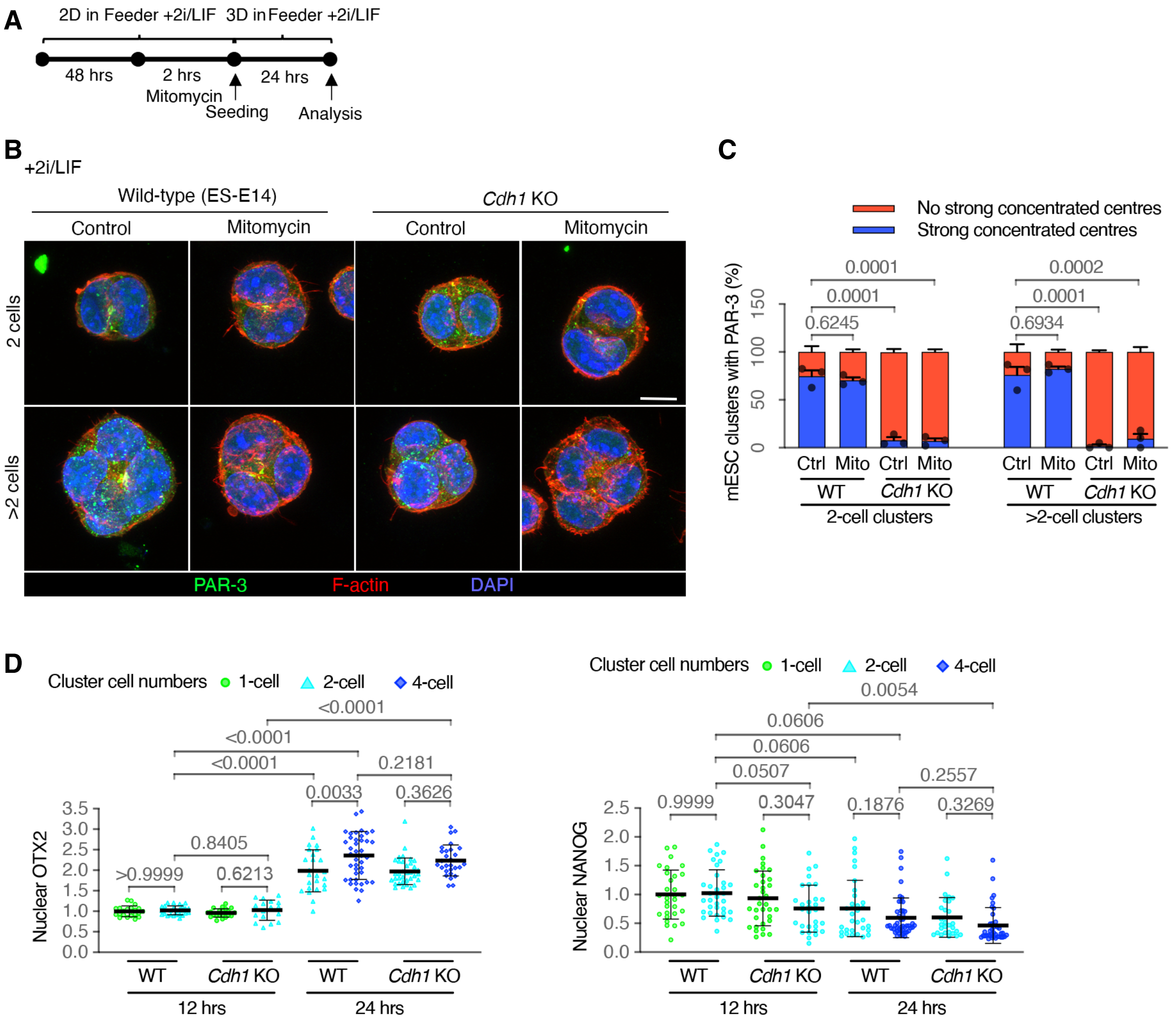

Figure EV3. AMIS seeding and pluripotency exit in wild-type and E-cadherin knockout mESCs.

A Timeline of experiment setups to assess *de novo* polarisation when mESCs were cultured with 2i/LIF to remain pluripotent.

B, C Immunofluorescence of PAR-3 (B) and quantification of the proportion of cell clusters with a polarised PAR-3 centre (C) in wild-type (ES-E14) and *Cdh1* knockout (KO) mESCs cultured in Matrigel for 24 h with 2i/LIF.

D Nuclear levels of OTX2 and NANOG based on immunofluorescence in wild-type and *Cdh1* KO mESCs at 12- or 24-h post-seeding into Matrigel. Only cells in interphase were analysed.

Data information: Data are presented as means ± SEM in (C); values of individual cells in dots and means ± SD in bars in (D). $n = 3$ experiments in (C), at least 20 clusters were analysed for each column in every experiment; 17–45 cells in each column from one experiment in (D). Two-way ANOVA analysis in (C) and (D); *P*-values were listed in the graphs. Scale bar: 10 µm.
Source data are available online for this figure.

 © 2022 The Authors

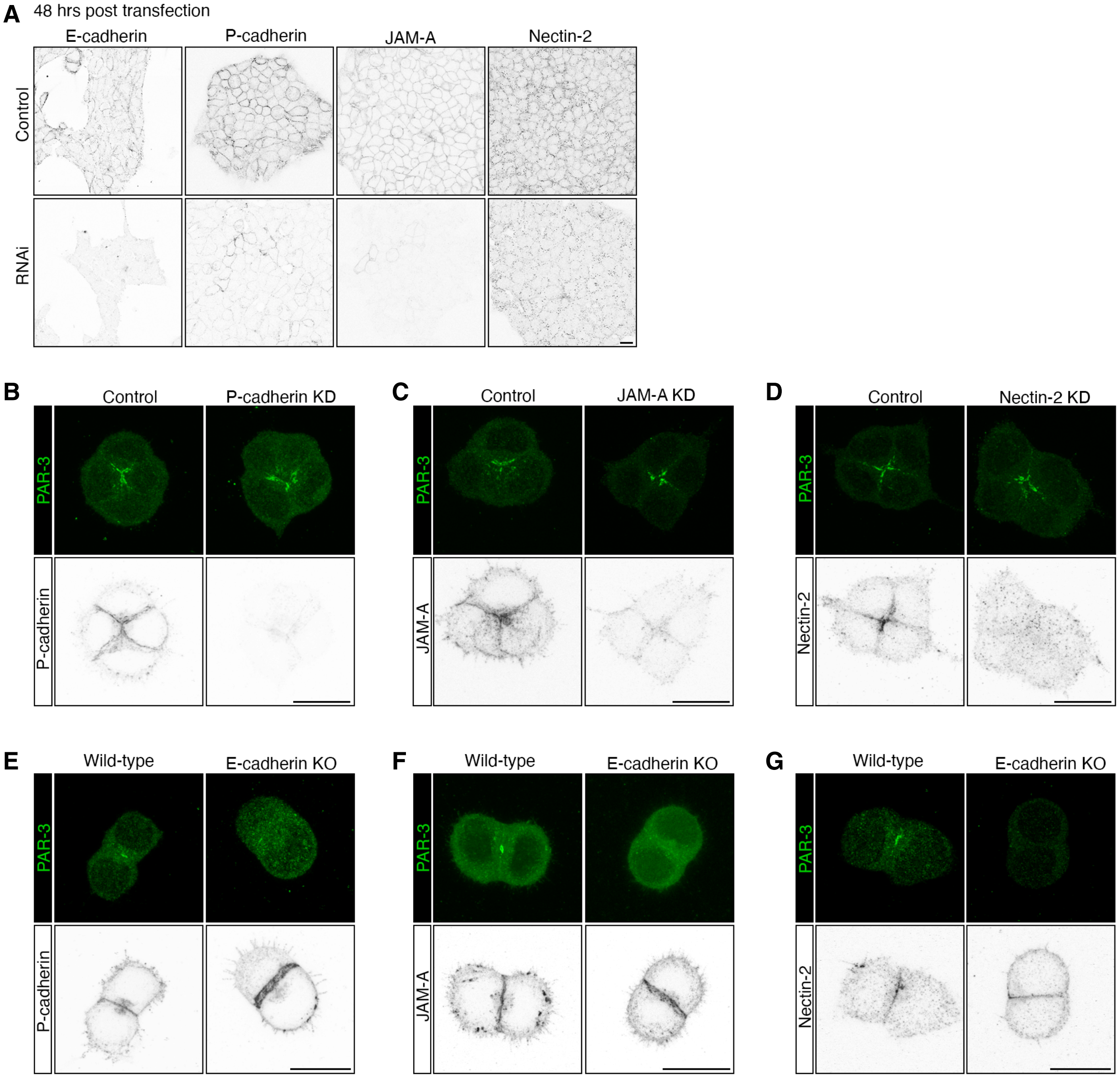

Figure EV4. Expression and knock-down of P-cadherin, JAM-A and Nectin-2 in mESCs.

A Expression and knock-down of E-cadherin, P-cadherin, JAM-A and Nectin-2 in mESCs (W4) cultured in 2D on gelatin. Scale bar: 15 μm.

B–D Expression of PAR-3 and P-cadherin (B), JAM-A (C) and Nectin-2 (D) in control and knock-down 4-cell mESC clusters cultured 24 h in Matrigel. Scale bars: 15 μm.

E–G Expression of PAR-3 and P-cadherin (E), JAM-A (F) and Nectin-2 (G) in wild-type (W4) and E-cadherin knockout (KO) mESC clusters cultured 24 h in Matrigel. Scale bars: 15 μm.

© 2022 The Authors

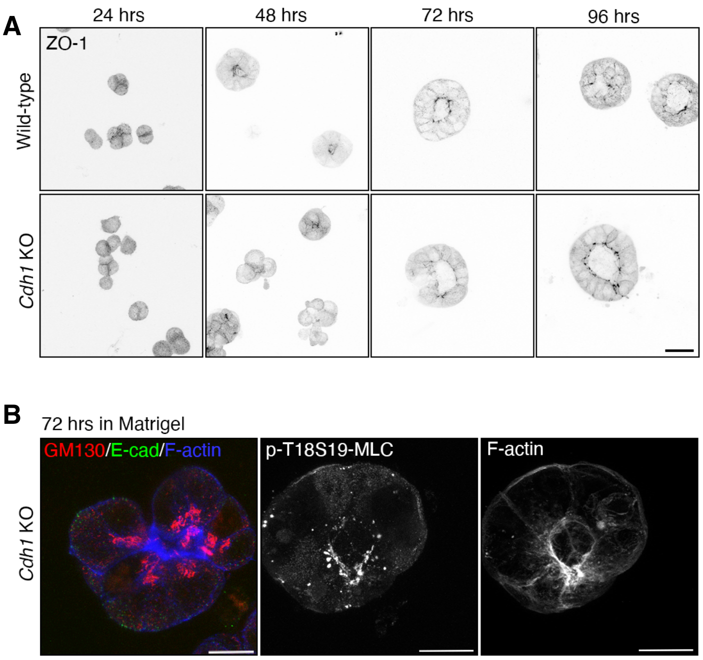

Figure EV5. Wild-type and E-cadherin knockout mESC cultured in Matrigel during lumenogenesis.

A ZO-1 immunofluorescence in wild-type (ES-E14) and E-cadherin knockout (*Cdh1* KO) mESCs cultured in Matrigel from 1–4 days. See Fig 6B for Podocalyxin staining. Scale bars: 25 µm.

B The Golgi network, phospho-myosin light chain 2 and F-actin in *Cdh1* KO mESCs cultured for 72 h in Matrigel. Scale bars: 25 µm.

 © 2022 The Authors
