# Supplementary material for: E‐cadherin mediates apical membrane initiation site localisation during de novo polarisation of epithelial cavities
Source: EMBO J. 2022 Aug 22;41(24):e111021. doi: 10.15252/embj.2022111021 (PMC9753465; doi:10.15252/embj.2022111021)
Supplement: Supplementary file 3 — Movie EV1 [file EMBJ-41-e111021-s010.zip › EMBOJ-2022-111021_MovieEV1/Movie_EV1_Legend.docx]

**Movie EV1 - Bright-field live movies of control and mitomycin-treated mESCs cultured in Matrigel.**

A, B Control mESCs cultured in Matrigel from 0 – 24 hours divided one (A) or two times (B).

C, D Mitomycin C treated mESC cultured in Matrigel from 0 – 24 hours did not divide but formed 2-cell clusters (C) or >2-cell clusters (D). Scale bar: 10 µm.
