# Supplementary material for: E‐cadherin mediates apical membrane initiation site localisation during de novo polarisation of epithelial cavities
Source: EMBO J. 2022 Aug 22;41(24):e111021. doi: 10.15252/embj.2022111021 (PMC9753465; doi:10.15252/embj.2022111021)
Supplement: Supplementary file 4 — Movie EV2 [file EMBJ-41-e111021-s007.zip › EMBOJ-2022-111021_MovieEV2/Movie_EV2_Legend.docx]

**Movie EV2 - Representative movies of mCherry-PAR6B in dividing and division-blocked mESCs cultured in Matrigel.**

A, B Control (A) and mitomycin division-blocked (B) mESCs cultured in Matrigel formed 2-cell doublets from 6 – 18 hours in Matrigel.

C, D Control (C) and mitomycin division-blocked (D) mESCs cultured in Matrigel formed multi-cell clusters from 9 – 19 hours in Matrigel. mCherry-PAR-6B localised to cell-cell contacts between 2 cells or the centre of multi-cell clusters after cell divisions (A, C) or after the cells touched (B, D). Scale bar: 10 µm.
