# Supplementary material for: E‐cadherin mediates apical membrane initiation site localisation during de novo polarisation of epithelial cavities
Source: EMBO J. 2022 Aug 22;41(24):e111021. doi: 10.15252/embj.2022111021 (PMC9753465; doi:10.15252/embj.2022111021)
Supplement: Supplementary file 5 — Movie EV3 [file EMBJ-41-e111021-s013.zip › EMBOJ-2022-111021_MovieEV3/Movie_EV3_Legend.docx]

**Movie EV3 - Representative movies of cysts forming in wild-type and E-cadherin knock-out mESCs cultured in Matrigel.**

Representative movies of the central 5µm z-stack of LifeAct-mRuby mESCs from control and cdh1 KO cell clusters as they make lumens.

A Wild-type mESCs cultured from 48 – 69 hours, frame interval = 1 hour.

B Cdh1 KO mESCs cultured from 48 – 68 hours, frame interval = 1 hour.

C Wild-type mESCs cultured from 74 – 83.5 hours, frame interval = 1 hour.

D Cdh1 KO mESCs cultured from 78 – 88.5 hours, frame interval = 30 min.

Scale bars: 25 µm.
