# Supplementary material for: E‐cadherin mediates apical membrane initiation site localisation during de novo polarisation of epithelial cavities
Source: EMBO J. 2022 Aug 22;41(24):e111021. doi: 10.15252/embj.2022111021 (PMC9753465; doi:10.15252/embj.2022111021)
Supplement: Supplementary file 6 — Movie EV4 [file EMBJ-41-e111021-s003.zip › EMBOJ-2022-111021_MovieEV4/Movie_EV4_Legend.docx]

**Movie EV4 - Rotation of the 3D rendering in Figure 6F.**

3D Rendering of wide-type (A) and E-cadherin knock-out (*Cdh1* KO, B) mESCs cultured in Matrigel. Central 5 µm of mESC cultures is shown at the end of the movie to better see the forming lumens. Scale bars: 10 µm.
